# Glaucoma Surgery: How Do We Get from Here to There?

**DOI:** 10.4103/0974-9233.56218

**Published:** 2009

**Authors:** Malik Y. Kahook

**Affiliations:** University of Colorado School of Medicine, Denver, CO, USA

This is an exciting time for those involved in the surgical care of glaucoma patients with new technologies being introduced at a dizzying rate. Procedures reviewed in this edition of MEAJO range from endocyclophotocoagulation which targets aqueous humor inflow to novel techniques targeting aqueous outflow such as canaloplasty.[1–2] While the guarded trabeculectomy remains the gold-standard intraocular pressure (IOP)-lowering procedure, the safety profile and lack of long-term success have opened the door for these newer surgeries to be explored and improved upon. It is clear to me that none of the newer techniques have replaced trabeculectomy in terms of IOP-lowering efficacy. The ideal surgical procedure for the broad range of diseases we collectively call glaucoma should combine the IOP-lowering results of antifibrotic enhanced trabeculectomy with more predictable and reproducible results. This intervention should be cost-effective and readily performed by practitioners of varying skill sets. It is hard to envision one procedure that might fulfill all of these criteria and perhaps a singular approach to such a complex disease is not the answer. So what is the answer and how do we get from here to there?

Physicians with experience managing post-trabeculectomy glaucoma patients will likely agree that not all blebs are created equal. The results after filtration surgery can vary widely between surgeons and even from case to case done by the same surgeon. The reasons for variability include differences in ocular anatomy, difficulty in creating a uniform opening into the anterior chamber, variability in scleral flap architecture and closure, unpredictable healing responses and poor wound modulation, non-adherence to postoperative anti-inflammatory regimens, and the list can go on and on. The introduction of aqueous drainage devices led to a degree of reproducibility from surgeon to surgeon and patient to patient by allowing for a uniformly sized connection to the anterior chamber and the lack of requisite scleral flap among other advantages. Still, variability in scarring as well as the inability to lower IOP to the level seen with trabeculectomy make it clear that the road to a new gold-standard surgery did not end with creation of these devices.

In an effort to bypass the limitations and complications involved with external drainage of aqueous humor to the subtenon's space, some of the newer devices attempt to restore natural outflow channels. The Glaukos istent (Glaukos Corp., Laguna Hills, CA) is one such device designed to bypass resistance to outflow, thought to be found in large part at the juxtacanalicular trabecular meshwork (TM), allowing for aqueous to reach the collector channels.[3] When combined with cataract extraction, this device appears to decrease IOP and has an appealing safety profile compared to more traditional surgeries. However, the IOP-lowering efficacy does not approach that of trabeculectomy and it appears that multiple devices are needed to achieve significant results. Another approach at improving flow through the conventional outflow system is known as canaloplasty (CP, iScience Interventional Menlo Park, CA).[2] This procedure involves the creation of a scleral flap and deep sclerectomy followed by threading a prolene suture through the canal of schlemm utilizing an elegant microcatheter. The suture is left behind and tightened to provide tension for 360 degrees, theoretically allowing for greater flow past the TM. The debate continues as to whether the IOP lowering in this procedure is due to tension on the TM or to the deep sclerectomy that would make this procedure very similar to that of viscocanalostomy. CP also has the potential to interfere with future trabeculectomy success due to scarring at the surgical site. Other devices addressing the outflow system are available, but all appear to decrease IOP to a similar level, still not approaching that of trabeculectomy. Perhaps it is a mistake to focus attention on the IOP-lowering efficacy of these procedures and deem them to be less desirable based on this standard.

**Figure 1:**
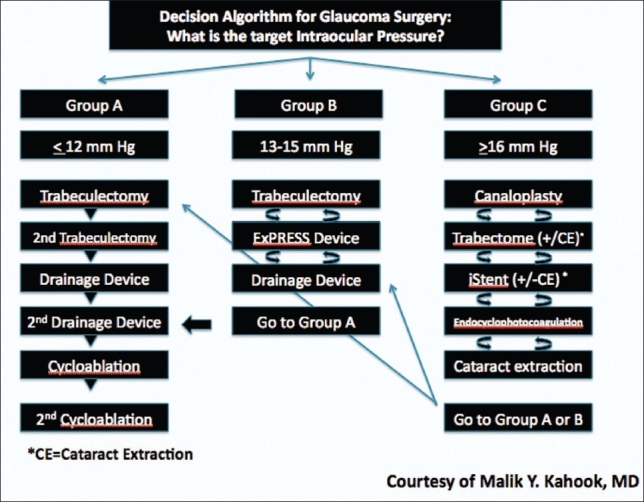
Decision algorithm for glaucoma surgery based on target intraocular pressure

I believe it is safe to say, from current published data, that focused alterations of the inflow and/or outflow systems will likely lead to safer surgeries but with limitations in the amount of IOP lowering achieved. While the debate rages on between which of the older or newer approaches to glaucoma surgery might be preferred, it might be more appropriate to better describe when to use each technique. It may be logical to use the newer techniques in earlier stages of glaucoma that might not require a very low target pressure. This would allow the treating surgeon to achieve success while avoiding complications and allowing for future access to tissues needed for trabeculectomy or drainage device surgery. Our decision tree for glaucoma care might look something like that depicted in Figure 1. For example, the surgery of choice for a patient with a target pressure of 16 mm Hg might first start with the Trabectome ab interno trabeculotomy (NeoMedix Inc, Tustin, California). If this procedure were to fail or if a lower target pressure is needed in the future, a trabeculectomy can be performed with no limitations. While a stepwise approach in this form is not a new idea, it is clear that we require more knowledge to determine where each procedure might fit best. Future studies will need to be done detailing outcome measures after successive procedures. The surgical algorithm can then be improved upon and perhaps even customized for different patient groups. The review articles in this edition detail the nuances behind many of the new techniques and give the reader insight into the pros and cons for each. It is now up to us to best decide how and when to use each of these procedures to tailor our care and improve patient outcomes.
